# Polarity-Dependent Driving Scheme for Suppressing Oil Film Splitting in Electrowetting Displays

**DOI:** 10.3390/mi16030338

**Published:** 2025-03-14

**Authors:** Jiashuai Wang, Xianyue Wu, Yibin Lin, Zichuan Yi, Mouhua Jiang, Yiting Rui, Liangyu Li, Li Wang, Xiuxiu Li, Liming Liu, Guofu Zhou

**Affiliations:** 1 School of Electronic Information, University of Electronic Science and Technology of China, Zhongshan Institute, Zhongshan 528402, China; 2 School of Integrated Circuit Science and Engineering (Exemplary School of Microelectronics), University of Electronic Science and Technology of China, Chengdu 611731, China; 3 Teaching Department of the Open University of China, Guangdong Open University (Guangdong Polytechnic Institute), Guangzhou 510091, China; 4 School of Information Engineering, Zhongshan Polytechnic, Zhongshan 511483, China; 5 School of Information Engineering, Guangzhou Panyu Polytechnic, Guangzhou 510006, China; 6 Guangdong Provincial Key Laboratory of Optical Information Materials and Technology, South China Academy of Advanced Optoelectronics, South China Normal University, Guangzhou 510006, China

**Keywords:** electrowetting displays (EWDs), driving scheme, oil film splitting, simulation model

## Abstract

Electrowetting displays (EWDs) face challenges such as oil film splitting and luminance fluctuations, hindering stable display performance. This study employed a high-precision three-dimensional simulation model to investigate and validate oil film splitting mechanisms. The model enabled detailed optimization of a new two-stage driving scheme, integrating a sinusoidal directing (SD) and a gradient asymmetrical alternating current (GAAC) driving scheme. The proposed scheme significantly suppressed oil film splitting, reduced luminance variance by 72.3% compared to traditional methods, and improved luminance stability by 41.6%. These findings highlight the potential of simulation-driven approaches to enhance EWD performance and expand applications of microfluidic technologies.

## 1. Introduction

Electrowetting display (EWD) is a new type of E-paper technology based on microfluidics [1,2,3], which not only offers the advantages of the previous generation of electrophoretic e-paper but also introduces technological advancements in video playback and full-color display [4]. Despite its advancements, EWDs still face challenges such as oil film backflow, low luminance, slow response speed, and luminance oscillation [5,6,7]. In response to these challenges, Shen et al. introduced a novel display system based on electro-microfluidic assembly, which could achieve high optical efficiency and precise color control by manipulating particle distribution in water-in-oil droplet arrays [8]. However, the system’s stability and responsiveness remain unoptimized, making it less mature than EWDs. Jiang et al. employed inkjet printing techniques to fabricate EWDs, which suppressed pixel structure distortion and reduced solid–liquid surface defects, thereby improving the luminance of EWDs [9]. Nevertheless, the problems of luminance oscillation and slow response speed remain to be addressed effectively. Recently, Lai et al. proposed a novel electrode structure for EWDs, which employed multiple electrode regions for pixels’ control [10]. However, this method made it difficult to maintain luminance stability during continuous display. Dai et al. proposed a new self-powered EWD system based on triboelectricity, which could achieve a colorful, dynamic display without an external power source [11]. However, its luminance stability and response speed were poor, limiting its usability for displaying high-quality images. Although these approaches have optimized certain aspects of EWD performance, they also increased costs and extended manufacturing cycles.

The splitting of the oil film leads to a decrease in the aperture ratio, which results in lower luminance under the same driving voltage. Additionally, during the prolonged driving process, the split oil film droplets are subjected to external vibrations, causing them to refuse and leading to irregular changes in the aperture ratio. This process weakens the display performance of EWDs. Furthermore, improper voltage selection during the design of a driving scheme can result in excessive pressure, potentially damaging the EWD. Given these limitations, we designed a high-precision three-dimensional simulation model for EWDs, which could analyze oil film splitting mechanisms and support the development of the driving scheme. Then, a new driving scheme, which was a method of controlling EWDs through voltage modulation [12], was proposed to mitigate luminance oscillations and improve display stability. The driving scheme was based on asymmetric design and polarity dependence to suppress oil film splitting at different stages of the display process, which was called the oil film splitting suppression (OFSS) driving scheme. By using this new driving scheme, the display performance of EWDs was improved.

## 2. EWDs Principles and Simulation Methods

### 2.1. EWDs Principles

**Figure 1:**
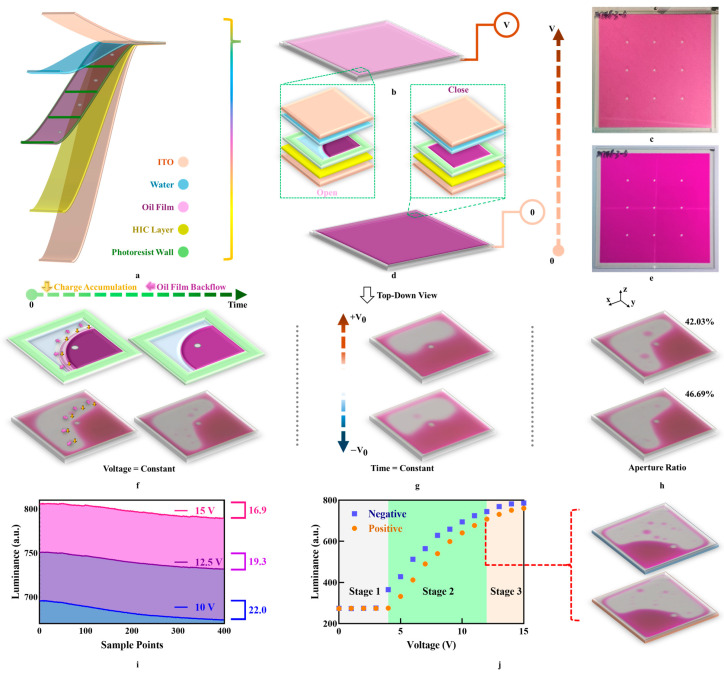
(**a**) Schematic diagram of the multilayer structure of EWDs. (**b**) Schematic of oil film contraction in a pixel when the EWD is turned on. (**c**) Photograph of the EWD in the opening state. (**d**) Schematic of oil film spreading in a pixel when the EWD is turned off. (**e**) Photograph of the EWD in the closing state. (**f**) Schematic and photograph of oil film backflow. (**g**) Photograph of polarity dependence. (**h**) Photograph of the aperture ratio. (**i**) The oil film backflow of different voltages. (**j**) Voltage–luminance curve, the points on the curve represent the luminance of the EWD driven by this voltage. The EWD pixel were recorded at two polar 12 V driving voltages which marked by red dot line.

The main structure of an EWD consists of multiple stacked layers of solid–liquid thin films [13,14], as shown in Figure 1a. The outermost layer is ITO glass, with a hydrophobic (HIC) layer coated on its underside. Photoresist barriers, created by photolithography, form pixel walls, which are filled with oil film [15]. A thin water film serves as a medium to assist the movement of oil film within pixel walls. Some pixels are equipped with a pillar to prevent pixels from deformation [16]. In summary, the EWD is essentially an optical switch. The opening and closing state of EWD is controlled by driving voltage.

First, when the EWD is not powered on, the oil film spreads on the pixel. As a result, the oil film color is displayed, and the pixel remains in the closing state, as shown in Figure 1d,e. When an appropriate driving voltage is used, the stable state of the oil film is broken and causes contraction which leads to an increase in the luminance of the EWD [17]. Once the applied driving voltage stops increasing and becomes a constant, the oil film–water interface reaches a relatively stable state, and the pixel switches to the opening state, as shown in Figure 1b,c. If the driving voltage is removed at this point, the pixel returns to the closing state. Therefore, the high-performance driving scheme based on driving voltage modulation can achieve fast response, high aperture ratio, and high stability. The relationship between the contact angle and the driving voltage is described by the Young-Lippmann equation [18].

In the driving process, charges continuously accumulate at the three-phase contact line (TPCL) among the oil film, water, and HIC layer until saturation is reached, generating a reverse voltage that reduces the contact angle [19,20]. It is a major factor in oil film backflow, as shown in Figure 1f. Oil film backflow can be weakened under high driving voltages, as shown in Figure 1i. The higher the driving voltage, the more obvious the oil film contraction and the shorter the three-phase contact line. Consequently, the reverse voltage which is generated by charge accumulation is inhibited.

Polarity dependence is the basic phenomenon of EWDs [21,22], which is manifested as different luminance under the same voltage with opposite polarity, as shown in Figure 1g. When designing an AC driving scheme, the different polarity driving voltage amplitude should be selected according to the absolute voltage–luminance curve, as shown in Figure 1j. This curve is divided into three stages. In stage 1, the EWD is still closed until the negative driving voltage is 3 V and the positive driving voltage is 4 V. The driving voltage at which the EWD is activated is referred to as the positive/negative threshold voltage. In stage 2, the EWD is in a safe driving stage. In stage 3, the contraction of the EWD approaches its maximum. Further increasing the voltage beyond this point may cause device breakdown, so driving voltages above 15 V are not recommended.

At the same time, polarity dependence not only impacts the activation threshold voltage but also affects the degree of oil film contraction, which is quantified by the aperture ratio. As the oil film ruptures into multiple droplets, the aperture ratio decreases [23,24]. This is because a sudden change in voltage will intensify the rupture of the oil film. Therefore, it is advisable to ensure a continuous voltage variation in the initial stage of the driving scheme. Because the measurement is complicated and positively correlated with luminance, the change in aperture ratio can be obtained indirectly by measuring the change in luminance, as shown in Figure 1h.

### 2.2. Simulation Methods

In a single-pixel model, the oil film was constrained to movement within the pixel, whereas water was permitted to flow freely above. Accordingly, the solid–liquid surface should be designated as a wetted wall condition, and the contact angle should be based on actual measurements. Furthermore, the water should be surrounded by an open boundary condition. Concurrently, a driving voltage was applied at the bottom of the model, and the top of the model was a grounded boundary condition. Additionally, the ITO glass could be disregarded in the model due to its minimal impact on the display effect.

To construct an accurate simulation model from partial differential equations (PDEs), it is essential to transform these equations into solvable algebraic equations through numerical discretization [25]. PDEs represent fundamental physical laws governing the behavior of the system, but they must be converted into computational models for practical simulation applications. In this study, the PDEs describing the electrowetting behavior of the EWD are discretized using the finite element method (FEM) for spatial resolution and the backward differentiation method (BDM) for time stepping [26]. This transformation enables numerical solvers to accurately compute the lower–upper decomposition of the matrix. Then, the dynamic opening and closing behavior in the EWD is solved. Proper boundary conditions and material properties were incorporated into the model to ensure the accuracy and reliability of the simulation results.

In laminar flow field methods, the motions of two-phase liquids could be described by the Navier–Stokes equation, as shown in Equation (1).

(1) $$\rho \frac{\partial v}{\partial t}+\rho \left(v∙\nabla \right)v=-\nabla p+\nabla ∙\left(\mu \left(\nabla v+{\left(\nabla v\right)}^{T}\right)-\frac{2}{3}\mu (\nabla ∙v)I\right)+F_{vf}+F_{st}$$

where ∙ is the inner product of variables, ρ is liquid density, v is liquid velocity, p is liquid pressure, and  μ is liquid dynamic viscosity; the surface tension and volume force $F_{st}$ and $F_{vf}$ are mainly caused by the surface tension and the driving voltage. Water and oil film are two completely immiscible, chemically non-reactive, incompressible, and Newtonian two-phase fluids [27]. In the EWD pixel structure, the fluid motion occurs at a low Reynolds number (Re ≪ 2000), indicating a predominantly laminar flow regime. Given the known incompressibility of the two-phase fluid in EWDs, the divergence of velocity ∇∙v is zero. So, the equation used in EWDs simulation is shown in Equation (2).

(2) $$\rho \frac{\partial v}{\partial t}+\rho \left(v∙\nabla \right)v=-\nabla p+\nabla ∙\left(\mu \left(\nabla v+{\left(\nabla v\right)}^{T}\right)\right)+F_{vf}+F_{st}$$

Furthermore, the external force $F_{st}$ can be described by the phase field method (PFM) from the chemical potential, and phase field variables are controlled the Cahn–Hilliard equation, as shown in Equation (3).

(3) $$\frac{\partial }{\partial t}\varphi_{p}+\left(v∙\nabla \right)\varphi_{p}=\nabla ∙\omega \nabla (\frac{\partial }{\partial \varphi }E_{t}-\nabla ∙\frac{\partial }{\partial \nabla \varphi }E_{t})=\nabla ∙\omega \nabla (E_{F})$$

where $\varphi_{p}$ is the scalar function used to distinguish between different liquids, ω is mobility, and $E_{t}$ is the total free energy density of the model, which could be described by the Ginzburg–Landau form, as shown in Equation (4).

(4) $$E_{t}=\frac{\lambda }{2}{\left|\nabla \varphi_{p}\right|}^{2}+\frac{\lambda }{4\varepsilon^{2}}{\left({\varphi_{p}}^{2}-1\right)}^{2}$$

where λ is the mixing energy density, and ε is a capillary width that scales with the thickness of the interface. Then, the $E_{F}$ can be described in Equation (5).

(5) $$E_{F}=\lambda \left[\nabla^{2}\varphi_{p}+\frac{\varphi_{p}\left({\varphi_{p}}^{2}-1\right)}{\varepsilon^{2}}\right]$$

Then, the Maxwell stress is added to the volume force $F_{vf}$ as the electric field force experienced by the per unit volume, as shown in Equation (6).

(6) $$F_{vf}=\nabla ∙M$$

Then, the force exerted on liquids within the pixel under voltage-driven conditions could be described by the Maxwell stress tensor M, as shown in Equation (7).

(7) $$M=\varepsilon_{0}\varepsilon_{r}\nabla \left[\begin{array}{ccc} E_{x}^{2}-E^{2}/2 & E_{x}E_{y} & E_{x}E_{z}\\ E_{x}E_{y} & E_{y}^{2}-E^{2}/2 & E_{y}E_{z}\\ E_{x}E_{z} & E_{y}E_{z} & E_{z}^{2}-E^{2}/2 \end{array}\right]$$

where $\varepsilon_{0}$ and $\varepsilon_{r}$ are the vacuum permittivity and the material permittivity, *M* is the Maxwell stress tensor, *E* is the electric field of the driving voltage, *E_x_*, *E_y_*, and *E_z_* are electric field components in *x*, *y,* and *z* directions.

The surface tension force $F_{st}$ is computed indirectly using the PFM via the Cahn–Hilliard equation. In this approach, the free energy of the system is defined in terms of the phase field variable, and its variational derivative—the chemical potential—is used to derive a source term that appears in the Navier–Stokes equations. In essence, the inhomogeneity of the phase field near the interface (quantified by its gradient) and the corresponding chemical potential jointly give rise to the surface tension force [6]. Therefore, $F_{st}$ is presented in Equation (8).

(8) $$F_{st}=(\frac{\varepsilon^{2}}{\lambda }E_{F}-\frac{\partial f}{\partial \varphi_{p}})\nabla \varphi_{p}$$

where $\frac{\partial f}{\partial \varphi_{p}}$ is the source of free energy. It not only provides the Maxwell stress but also changes the contact angle at the solid–liquid contact surface, as shown in Equation (9).

(9) $$\theta_{V}=\cos^{-1}\left(\cos\theta_{0}+\varepsilon_{0}\varepsilon_{r}V^{2}/2\gamma d\right)$$

where $\theta_{V}$ is the contact angle under an applied driving voltage, $\theta_{0}$ is the contact angle without applied driving voltage, γ is the interfacial tension between two liquids, and *d* is the thickness of the HIC layer. When the EWD is driven for a long time, charges accumulate at the TPCL, and the condition of interface charge accumulation is added to the model, as shown in Equation (10).

(10) $$\sigma_{q}=\int nJ_{t}dt$$

where $\sigma_{q}$ is the surface charge density, $nJ_{t}$ is the normal component of the total current density. In the three-dimensional single-pixel simulation model, the total current density is mainly in the *z* direction. The reverse voltage can be described by the capacitance–voltage equation, as shown in Equation (11).

(11) $$V_{r}=\frac{\int \sigma_{q}ds}{C_{p}}$$

where $V_{r}$ is the reverse voltage and $C_{p}$ is the capacitance of a single pixel. Therefore, the contact angle is reformulated in Equation (12).

(12) $$\theta_{V}=\cos^{-1}\left(\cos\theta_{0}+\varepsilon_{0}\varepsilon_{r}{\left(V-V_{r}\right)}^{2}/2\gamma d\right)$$

Therefore, the contact angle at the solid–liquid interface in the three-dimensional model is set to $\theta_{V}$. Then, the application of Naiver slip condition and interface charge accumulate condition can enhance the precision of the model. There is a viscous effect on the solid–liquid contact interface. Tangential stresses can be added to the model to simulate the viscous effects of liquid motion [28]. The tangential stress is shown in Equation (13).

(13) $$K_{ts}=-\frac{\mu }{\beta }u$$

where $K_{ts}$ is tangential stress, u is liquid velocity tangential to the surface, and  β is a slip length. A Naiver slip condition is used in the model to simulate the viscous effects.

## 3. Oil Film Splitting Simulation

**Figure 2:**
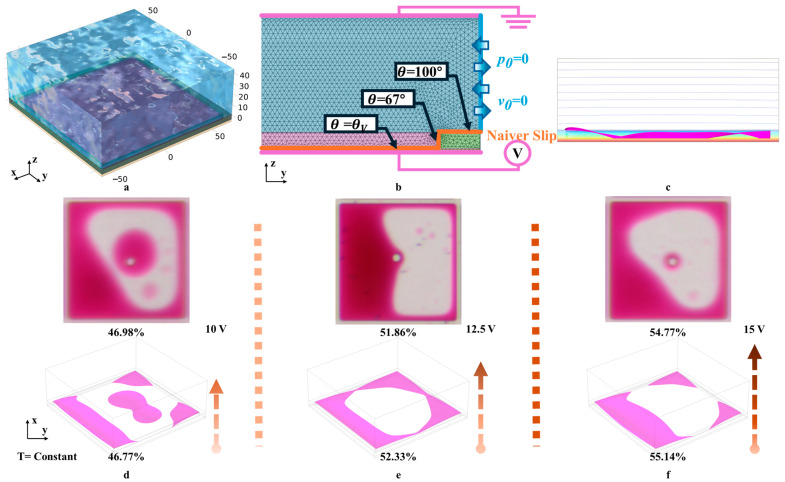
(**a**) Schematic diagram of the high-precision 3D single-pixel simulation model. The simulation model was symmetrical in terms of the xoz and yoz planes. (**b**) The setting of boundary conditions. Purple represented the boundary conditions of the electrostatic field. Blue represented the boundary conditions of the entrance and exit. Orange represented the wetting wall boundary conditions. (**c**) The cross-sectional view of oil film and electric field. (**d**) Experiment and simulation results at V_0_ = 10 V. (**e**) Experiment and simulation results at V_0_ = 12.5 V. (**f**) Experiment and simulation results at V_0_ = 15 V.

Based on material and physical parameters, a high-precision three-dimensional simulation model was established by us. It was assumed that the liquid temperature (25 °C) remained constant during motion, so the thermal expansion of the liquid was negligible. Furthermore, gravity and pressure at the micron scale were significantly smaller than surface tension, allowing the effects of pressure and gravity to be disregarded. In addition, due to the constant temperature and the neglect of gravity and pressure, the dynamic viscosity of the liquid was set to a fixed value. The liquid’s response to polarization at a low frequency was primarily characterized by the free rotation of molecules, and its relative dielectric constant did not significantly change with variations in driving voltage frequency, allowing it to be treated as a constant. The oil film height was set to correspond to the pixel wall height, as shown in Figure 2a. The parameters used in this model are shown in Table 1.

Because the model was used to simulate the single gray level luminance, it was acceptable that the contact angle in this model was set to a constant. Therefore, contact angle hysteresis had little effect on simulation [29,30]. Solid–liquid contact surfaces were defined as wall boundary conditions which restricted the liquid’s domain of motion, meaning that the velocity component normal to the wall was set to zero. Water could flow freely laterally, subject to inlet/outlet conditions. Initially, the water remained stationary, so the initial pressure was set to $p_{0}$ = 0, and the initial velocity was set to $v_{0}$ = 0. The solid–liquid contact surface was defined as a wetted wall boundary condition, and the contact angle for each wetted wall was configured based on the structural and material properties of the EWD. In the electrostatic field, charge conservation boundary conditions were applied based on the dielectric model of relative permittivity. The bottom and top outer boundaries of the model were assigned potential and ground boundary conditions, respectively, while the remaining outer boundaries were given zero charge boundary conditions, as shown in Figure 2b.

The model was used to test the effects of different voltages on oil film splitting and electric fields caused by the driving voltage, which could analyze oil film splitting mechanisms and support the development of the driving scheme. The simulation process of oil film motion and electric field are shown in Figure 2c. Through simulation models, EWD destruction caused by incorrect selection of driving voltage could be avoided. Additionally, the model could predict the movement pattern of the oil film, providing a foundation for precise control of the oil film. To prevent the destruction caused by the oil film from going over the wall, the DC driving voltage was set to 10 V, 12.5 V, and 15 V, and the simulation results are shown in Figure 2d–f. When pixels were driven by a lower voltage, the oil film splatted, and droplets formed in the pixel center, as shown in Figure 2d. So, it was difficult to hold together, resulting in a reduced aperture ratio of 46.77%. When pixels were driven by a higher voltage, the EWD could obtain a higher aperture ratio of 55.14%, but the oil film overflow might occur, as shown in Figure 2f. However, when the voltage exceeds 15 V, the EWD may be damaged. These findings suggest that the driving voltage range should be limited from 10 V to 15 V to balance the aperture ratio and stability, as shown in Figure 2e.

Meanwhile, the oil film contraction morphology within pixels under different driving voltages was captured, as shown in Figure 2d–f. It was observed that the aperture ratio of pixel units from actual tests closely matched that obtained from the simulation model, with a difference in aperture ratio of less than 0.5%. Consequently, the simulation results accurately reflected the movement morphology of the oil film.

## 4. Driving Scheme

**Figure 3:**
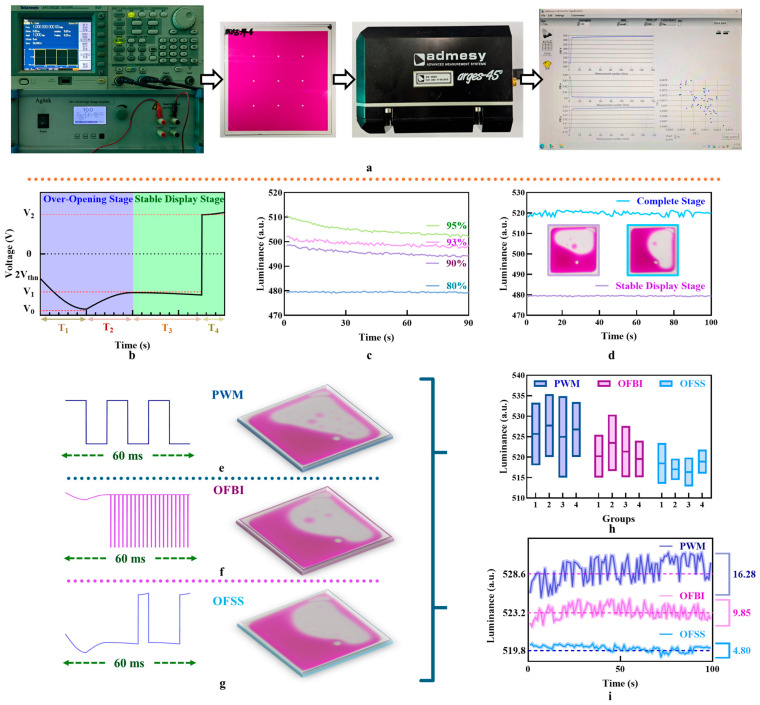
(**a**) Experimental platform diagram. (**b**) Schematic of the pixel simulation model. The duration of the driving scheme in this figure was 40 ms (**c**) Comparison of different duty cycles under the GAAC driving scheme. (**d**) Display performance comparison between the complete stage and the stable display stage. (**e**) Schematic of the PWM driving scheme and the pixel performance [22]. (**f**) Schematic of oil film backflow inhibition driving scheme and the pixel performance [23]. (**g**) Schematic of the proposed driving scheme and the pixel performance. (**h**) Luminance–time curve of EWDs under three driving schemes. (**i**) Long-term testing results of multiple groups of EWDs under three driving schemes.

An experimental platform was set up to test the luminance–time curve of the EWD. First, the driving scheme file was imported into a generator. The generator then converted the driving scheme into signals to control the voltage output, which was subsequently sent to a voltage amplifier. The voltage amplifier then adjusted the driving voltage to appropriate levels, thereby enabling the operation of the EWD. Subsequently, the change in luminance of the EWD was measured using a colorimeter. Finally, the measurement data were imported into a computer for sorting, as shown in Figure 3a.

Grounded in these principles and the simulation model, the proposed driving scheme in this paper was divided into two stages: an over-opening stage and a stable display stage. These stages were designed to mitigate issues such as oil film splitting and overflow, while optimizing aperture ratio and stability. The over-opening stage employed an SD driving scheme, and the stable display stage utilized a GAAC driving scheme, as shown in Figure 3b. In each stage, the total driving time was 20 ms, which was sufficient to support 50 Hz video playback for dynamic display requirements. This division ensured smoother transitions and controlled voltage application during EWD operations.

During the over-opening stage, the SD driving scheme was further divided into two stages: a steep increasing stage and a slow descending stage. The purpose of this division was to achieve a higher aperture ratio. In the steep increasing stage, the initial driving voltage increased from twice the negative threshold voltage *V_thn_* to the maximum amplitude *V*_0_, and the duration was *T*_1_ = 10 ms. This gradual increase mitigated oil film splitting, a common issue caused by abrupt voltage fluctuations, while ensuring sufficient contraction of the oil film. Importantly, *V*_0_ was set higher than the stabilized voltage *V*_1_ to enhance the aperture ratio, even though the initial driving voltage was lower than *V*_1_. Then, in the slow descending stage, the driving voltage decreased from *V*_0_ to *V*_1_, the duration of which was *T*_2_ = 10 ms. The reason a relatively small *V*_1_ could maintain consistently high luminance was that it had to overcome static friction before it could move again when the oil film remained fully contracted. It ensured that the oil film stayed in its fully contracted state, achieving a stable luminance. During this stage, the oil film reached the target contraction required for optimal display performance. To sum up, the voltage–time equation of the SD driving scheme was described by Equation (14).

(14) $$V_{st1}=\left\{\begin{array}{cc} 2V_{thn}+k_{1}\sin\left(\frac{\pi t}{{2T}_{1}}\right) & t\in \left(0,10\right)\\ V_{0}+k_{2}\sin\left(\frac{\pi t}{{2T}_{2}}\right) & t\in \left(10,20\right) \end{array}\right.$$

where *V_st_*_1_ was the real-time voltage for the over-opening stage, t was time, *T*_1_ and *T*_2_ were driving times, and *k*_1_ and *k*_2_ were constants to ensure that the driving scheme was continuous. At the end of this stage, the oil film was highly contracted to achieve a high luminance. Then, the GAAC driving scheme with a stable display stage and a charge release stage was employed for a long-term display in the stable display stage. The voltage–time equation of the GAAC driving scheme was described by Equation (15).

(15) $$V_{st2}=\left\{\begin{array}{cc} V_{1}+k_{3}\sin\left(\frac{\pi t}{2T_{3}}-\frac{\pi }{2}\right) & t\in \left(0,T_{3}\right)\\ V_{2}+k_{4}\sin\left(\frac{\pi t}{{2T}_{4}}-\frac{\pi }{2}\right) & t\in \left(T_{3},20\right) \end{array}\right.$$

where *V_st_*_2_ was the real-time voltage for the stable display stage, *V_st_*_2_ was introduced during the stabilized driving stage, which lasted for *T*_3_, *V*_2_ was introduced during the charge release stage, which lasted for *T*_4_. The total duration of *T*_3_ and *T*_4_ was 20 ms. A gradual increase in driving voltage was designed at the end of the stabilized driving stage and during the charge release stage to optimize performance. This is because it took time for polarity switch of the voltage, resulting in a very short low driving voltage stage. At this time, the aperture ratio decreased, resulting in a decrease in luminance. To suppress this situation, the driving voltage was increased at the end of each stability driving stage and the charge release stage to increase the aperture ratio.

At the same time, the optimal time duty cycle of *T*_3_ and *T*_4_ during the stable display stage was determined, as shown in Figure 3c. When the duty cycle was 95%, the luminance decreased 1.76%, oil film backflow could not be suppressed. The best suppression of oil film backflow could be obtained when the duty cycle of *T*_3_ was 80%, with a corresponding luminance of 479.8. Therefore, the duty cycle of the GAAC driving scheme was set to 80% in the stable display stage. Additionally, as shown in Figure 3d, the time-luminance curve was tested to observe the EWD’s transition from the closing state to the target luminance under the driving scheme with two complete stages. It was observed that the over-opening stage effectively increased luminance.

Finally, the OFSS driving scheme was compared with the traditional pulse-width modulation (PWM) driving scheme and the driving scheme of oil film backflow inhibition (OFBI) [22,31]. These driving schemes were tested using parameters recommended in this paper, as shown in Table 2.

When the EWD was driven by the PWM driving scheme, as shown in Figure 3e, the pixel’s aperture ratio was high. Photos of pixels in the stable display stage were taken by a microscope. The average luminance was 528.6, and the split oil film droplets were marked in the red cycle. However, there was a significant occurrence of oil film splitting, which resulted in an unstable display performance, with the average luminance variance of 4.87, meanwhile, when the EWD was driven by the OFBI driving scheme, as shown in Figure 3f. The average luminance was 520.6, and the split oil film droplets were marked in the red cycle. In this case, oil film splitting was weakened, and the average luminance variance was 2.31. In contrast, when the EWD was driven by the OFSS driving scheme, as shown in Figure 3g, the average luminance was 516.9, oil film splitting was effectively suppressed, and the average luminance variance reduced to 1.35. Hence, the driving scheme proposed in this paper resulted in a 2.2% reduction in luminance compared to the PWM driving scheme. This decrease in luminance occurred because the optimal duty cycle of 80% was necessary to effectively suppress oil film backflow, which limited the achievable luminance to 516.9. However, this adjustment significantly reduced luminance fluctuations, thereby enhancing overall display stability.

The luminance–time curve was shown in Figure 3h. and the total driving time was 100 s. The OFSS driving scheme exhibited the lowest display luminance but provided the most stable display performance. Additionally, the luminance fluctuation between adjacent sampling points was minimal, preventing screen flickering, which could be detected by the human eye. The repeatability of the OFSS driving scheme was also good in repeated tests across multiple groups, the maximum luminance fluctuation was only 10.62. This fluctuation was reduced by 47.86% compared to the PWM driving scheme, as shown in Figure 3i. Furthermore, when the EWD was driven by the proposed driving scheme, the luminance stability was improved by 41.6% compared to the OFBI driving scheme, with the average maximum luminance fluctuation of 7.1. At the end of all experiments, EWDs were not damaged by the excess voltage from the proposed driving scheme.

## 5. Discussion and Conclusions

In conclusion, the high-precision three-dimensional simulation model presented in this paper validated its effectiveness for observing oil film morphology and oil film splitting over short time intervals. The oil film splitting and oil overflow were simulated using this model, which also played a key role in optimizing the proposed driving scheme. Based on polarity dependence, this scheme effectively suppressed oil film splitting and enhanced the display stability of EWDs. Furthermore, the combination of simulation models and experimental methods effectively verified the reliability of various driving schemes, broadening the research approaches for EWD development. In addition, as an application scenario of microfluidic principles, the research of EWDs in this article can be extended to the field of simulation and driving of microfluidic research.

## Figures and Tables

**Table 1:** The parameters used in this model.

Parameters	Values	Units
Oil Film Density	0.880	g/cm^3^
Water Density	0.998	g/cm^3^
Oil Film Dynamic Viscosity	0.004	Pa× s
Water Dynamic Viscosity	0.001	Pa× s
Oil Film Relative Dielectric Constant	4.1	1
Water Relative Dielectric Constant	79	1
HIC Layer Dielectric Constant	1.3	1
Photoresist Wall Dielectric Constant	3.3	1
Pixel Total Height	50	μm
Oil Film Height	6	μm
Water Height	42	μm
HIC Layer Height	2	μm
Photoresist Wall Height	6	μm
Photoresist Wall Width	15	μm
Pixel Total Area	160×160	${\mu m}^{2}$

**Table 2:** The luminance data of EWD was driven by driving schemes.

Driving Scheme	PWM	OFBI	OFSS
Min Luminance	518.28	515.44	514.15
Average Luminance	528.06	520.39	516.93
Max Luminance	534.28	526.87	521.20

## Data Availability

The original contributions presented in this study are included in the article. Further inquiries can be directed to the corresponding author.
